# Physiological and performance effects of a simulated prolonged service game in competitive male tennis players

**DOI:** 10.5114/biolsport.2026.160854

**Published:** 2026-05-20

**Authors:** André V. Brito, Filipa Cardoso, Anna Ogonowska-Slodownik, Alejandro Javaloyes, Jaime Fernandez-Fernandez, Ricardo J. Fernandes

**Affiliations:** 1 Centre of Research, Education, Innovation and Intervention in Sport (CIFI2D) and Porto Biomechanics Laboratory (LABIOMEP), Faculty of Sport, University of Porto, Porto, Portugal; 2 Faculty of Rehabilitation, Jozef Pilsudski University of Physical Education in Warsaw, Warsaw, Poland; 3 Center of Sports Research, Miguel Hernández University of Elche, Elche, Spain; 4 Faculty of Physical Activity and Sports Sciences, Universidad de León, León 24004, Spain; 5 AMRED, Human Movement and Sports Performance Analysis, Universidad de León, 24007 León, Spain

**Keywords:** Fatigue, Anaerobic metabolism, Electrolyte balance, Neuromuscular coordination, Motor performance

## Abstract

This study investigated the acute physiological and performance responses to a simulated prolonged service game designed to replicate the most demanding phases of competitive tennis. Fourteen nationally ranked male players (17.8 ± 0.9 y) performed 12 consecutive maximal intensity serves, each immediately followed by eight alternating forehands and backhands. Internal load was monitored before, during and after the protocol using heart rate, oxygen uptake and venous blood samples (ammonia, creatine kinase, albumin, sodium, calcium, lactate and glucose). External load was quantified with a Doppler radar gun to measure ball speed and high-frame-rate video to assess serve precision. Statistical significance was set at *p* ≤ 0.05. Plasma concentrations of ammonia and creatine kinase (both *p* < 0.01), albumin, sodium, calcium (all *p* < 0.05), lactate (*p* < 0.01) and glucose (*p* < 0.05) increased from baseline to post-protocol. Heart rate and oxygen uptake rose rapidly in the initial phase and stabilized at ~85 and 62% of maximal values, respectively. Serve speed and precision began to decline from the sixth point onward, with reductions of 5.4 and 29%, coinciding with peak lactate and ammonia values. A prolonged service game imposes considerable anaerobic-metabolic, cardiovascular, and neuromuscular stress on competitive tennis players, producing measurable impairments in technical performance well before task completion. Systematic monitoring of blood metabolites and electrolytes can help coaches individualize recovery intervals and design conditioning programs that enhance players’ tolerance to repeated high-intensity serving, thereby preserving technical consistency under competitive fatigue conditions.

## INTRODUCTION

Tennis is a high-intensity intermittent sport characterized by repeated explosive actions, including serves, forehands, backhands and short-distance sprints (3–12 m) [1]. These actions are predominantly fueled by anaerobic energy systems, while aerobic metabolism contributes during brief recovery intervals between points, facilitating phosphocreatine resynthesizes and the clearance of metabolic byproducts [2]. Points typically last 5–40 s, interspersed with 20–30 s of rest between points and 60–90 s between games, resulting in match durations of 1–3 h [3]. Match demands are further modulated by environmental conditions, court surface and players’ fitness levels [4], which influence point length and associated physiological stress [5]. In addition, age and maturational stage may affect tennis physical capacities and performance, particularly in younger competitive players [6]. Recent investigations highlight the interplay between anaerobic and aerobic metabolism in sustaining performance and optimizing recovery during prolonged matches [7].

During a match, heart rate and oxygen uptake commonly reach ~70–80% and 50–60% of maximal values (respectively), while blood lactate concentrations typically range from 2–4 mmol · L^−1^, reflecting the anaerobic demands of high-intensity points [7]. Under conditions of prolonged or repeated points, heart rate, oxygen uptake, and blood lactate (often exceeding 8 mmol · L^−1^) progressively increase, indicating greater reliance on anaerobic metabolism [8]. The Borg 6–20 scale is widely applied in tennis to assess perceived exertion, showing strong relations with physiological markers such as lactate concentration [9]. Low-intensity efforts are generally rated 12–13, whereas extended high-intensity rallies are scored 15–20 [10]. Integrating subjective measures of exertion with objective physiological data enhances the precision of training load monitoring and facilitates individualized conditioning programs [11, 12].

Tennis performance is also influenced by unpredictable factors such as match duration, tactical variability and environmental conditions, which require the integration of physical, technical, tactical and psychological domains [7, 13]. Repeated high-intensity and prolonged points induce fatigue [10], characterized by elevated lactate and ammonia levels that impair acid–base balance and neuromuscular coordination. When these demands exceed the body’s ability to maintain homeostasis, glycolytic activity intensifies, leading to hydrogen ion accumulation, pH reduction and metabolic acidosis [14]. This stress is compounded by disturbances in glucose and fatty acid metabolism and by electrolyte imbalances (e.g. potassium, sodium and calcium), which reduce muscular efficiency and coordination [12]. Such physiological perturbations may compromise neuromuscular control and cognitive function during decisive game moments [15].

The serve, the action initiating every point, is particularly vulnerable to fatigue due to its biomechanical complexity and reliance on precise neuromuscular coordination. Prolonged points exacerbate metabolic and neuromuscular stress [16], reducing energy availability and inducing a fatigue state [17]. This leads to decrements in muscle strength, coordination and cognitive speed, impairing serve mechanics, technical execution and movement efficiency. Neuromuscular deficits are especially evident during fast serves, where joint instability and mistimed muscle activation disrupt kinetic chain dynamics, reducing ball speed and precision [10]. Given the tactical importance of serve speed in modern tennis [18], such impairments can be decisive. Fatigue also slows motor responses and affects decision-making [19], further undermining serve effectiveness under pressure [20].

Monitoring physiological biomarkers such as oxygen uptake, heart rate and blood lactate provides essential insights into players’ physiological load and recovery dynamics during tennis specific training and simulated match conditions [21]. This information helps coaches adjust training loads, recovery intervals and match strategies, minimizing overload and injury risk [22]. Despite growing interest in tennis physiology, few studies have examined fatigue responses during tennis matches. A recent review identified limited research on key physiological variables, with only three studies evaluating heart rate during serve performance [15, 18]. This study aimed to examine the acute physiological and serve performance responses of tennis players during and after a standardized and simulated prolonged service game. The protocol was designed to simulate highly demanding phases of a match through repeated maximal intensity serves followed by consecutive shots, with emphasis on fatigue effects on serve speed and precision. It was hypothesized that fatigue would be associated with changes in physiological indicators and impairments in serve execution.

## MATERIALS AND METHODS

### Subjects

Fourteen competitive male tennis players volunteered to participate in this study (17.8 ± 0.9 years, 179.3 ± 5.8 cm, 68.4 ± 8.1 kg and 15.1 ± 5.1 h of weekly training). Participants had more than 10 years of structured tennis practice and competed in 20.7 ± 5.7 national and/or international tournaments per season [23]. All players were nationally ranked within the top 50 in their respective age categories. Anthropometric measurements (dominant vs non-dominant limbs) were taken to characterize players’ physical profiles, including limb circumferences (cm): relaxed biceps (27.3 ± 2.5 vs 26.2 ± 2.3), flexed biceps (29.8 ± 2.6 vs 28.5 ± 2.3), forearm (26.5 ± 1.6 vs 25.3 ± 1.5) and wrist (16.5 ± 0.7 vs 16.3 ± 0.6). Additional circumferences were recorded for the chest (88.3 ± 6.0), waist (74.6 ± 3.8), hips (94.1 ± 4.3), thigh (50.9 ± 4.3), calf (38.9 ± 13.3) and ankle (23.5 ± 3.5). Subcutaneous fat was assessed via skinfold measurements (mm) at eight standard anatomical sites: triceps (10.8 ± 4.7), biceps (9.8 ± 2.7), iliac crest (6.0 ± 2.1), subscapular (15.9 ± 4.9), suprailiac (6.8 ± 2.2), abdominal (11.3 ± 3.4), thigh (13.5 ± 5.3) and calf (9.0 ± 3.7).

Inclusion criteria were: Participants were male competitive tennis players aged 15–20 years, nationally ranked within the top 50 in their respective age categories and actively competing in national and international tournaments. Exclusion criteria included chronic or acute health conditions, history of severe joint or muscle injuries, and current use of medication that could affect performance. The study was approved by the local ethics committee (CEFADE 05.2022) and conducted in accordance with the Declaration of Helsinki. Written informed consent was obtained from all participants or, in the case of minors, from their parents or legal guardians.

### Procedures

**FIG. 1:**
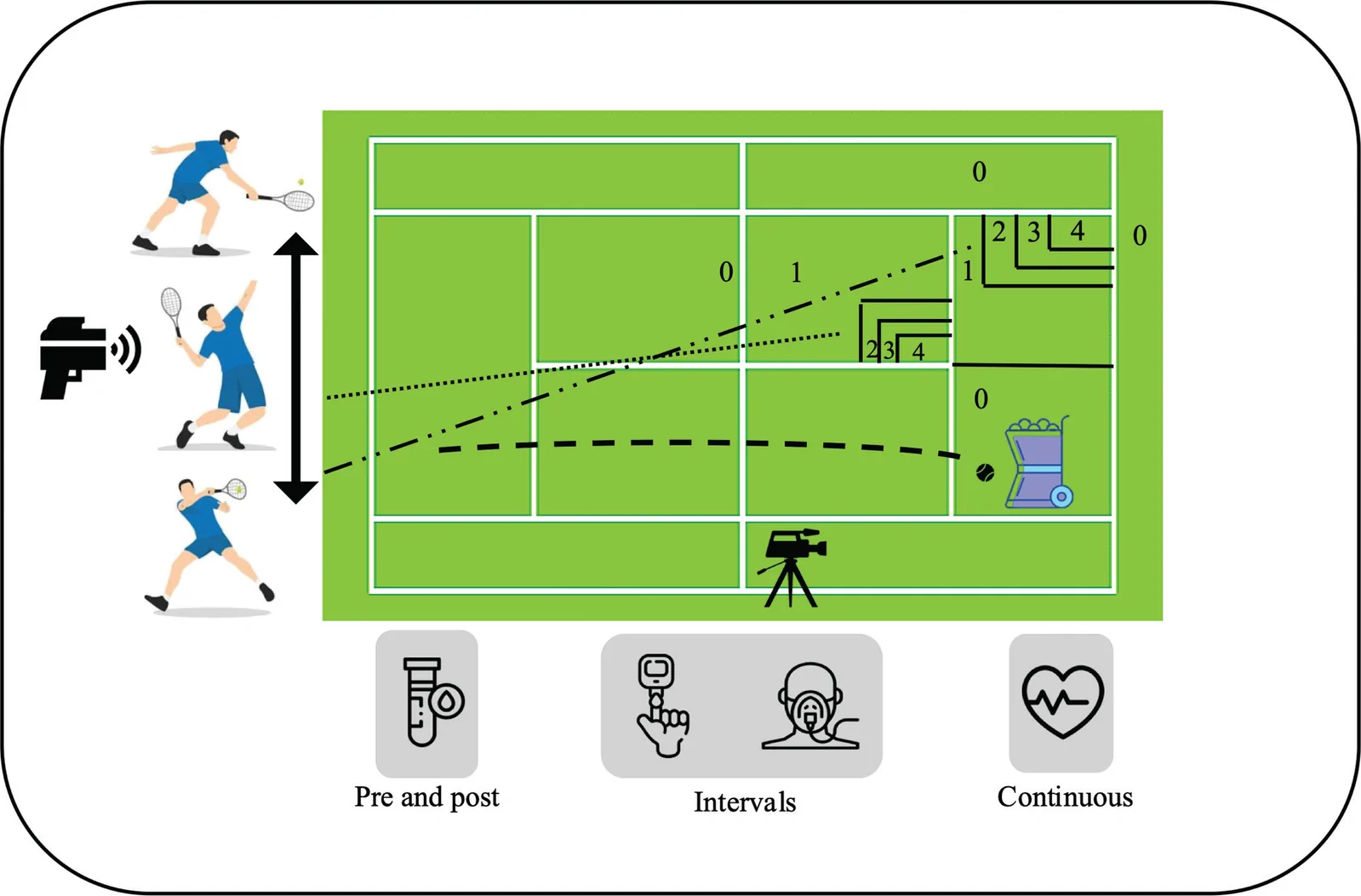
Experimental setup for the prolonged service game protocol. Numbers 1, 2 and 3 correspond to the flat serve, forehand and simulated backhand (respectively), with precision values indicated for each target area. Data collection moments (pre and post protocol, during rest intervals and continuous monitoring) and the equipment used for measuring serve speed and precision are also shown.

Each player completed a simulated prolonged service game protocol on an outdoor grass court. After a standardized 15 min warm-up consisting of agility exercises, tennis-specific drills and progressively faster serves, players executed 12 consecutive maximal-intensity simulated points. Each point began with a flat serve followed by eight forehands and eight backhands performed from a square stance (lower limbs parallel and lateral to the net; Figure 1) [17]. The protocol was designed to induce progressive physiological and neuromuscular fatigue under controlled conditions and was not intended to replicate a typical point duration on grass courts. Players used their own rackets, and new tennis balls were delivered from a baseline ball machine (Slinger Bag, Windsor Mill, MD, USA) at 100 km · h^−1^ with 4 s between balls [24], which produced a standardized topspin trajectory that ensured consistent ball flight characteristics throughout the repetitions. Serve speed was recorded using a Doppler radar gun positioned 2 m behind the player (Stalker Pro II, Richardson, TX, USA). Serve precision was quantified using a 240 Hz highspeed camera (GoPro HERO6, San Mateo, CA, USA) with three predefined target areas: large (2.25 m^2^), medium (1.00 m^2^) and small (0.25 m^2^) [20].

Prior to and immediately after the protocol, 100 *µ*L of capillary blood was collected into heparinized tubes and analyzed with the Piccolo Xpress^®^ system (Abaxis, Union City, CA, USA) using a comprehensive metabolic panel disk (AmLyte 13). Each self-calibrating disk contained an internal barcode that uploaded calibration constants and quality-control data to the analyzer [25]. Fourteen biochemical variables were assessed: alanine aminotransferase, albumin, amylase, aspartate aminotransferase, calcium, C-reactive protein, creatine kinase, creatinine, glucose, potassium, sodium, total bilirubin and blood urea nitrogen. Built-in control wells ensured analytical reliability by verifying reagent integrity, sample adequacy, optical performance and possible interference from lipemic, hemolyzed or icteric samples [25]. After placement in the analyzer, each sample was rotated to separate the plasma, mixed with diluent in the mixing chamber and analyzed, with results displayed after 12 min.

Ammonia concentration was determined from an additional 20 *µ*L capillary blood sample using a PocketChem BA analyzer (Menarini Diagnostics, Florence, Italy) [26]. The device was calibrated at the start of each testing day and after every tenth sample to ensure analytical stability and accuracy. Blood samples were alkalinized in borate buffer, allowing gaseous ammonia to diffuse through a semipermeable membrane and react with a bromocresol-green indicator strip. After a 180 s incubation, the strip was inserted into the reading device and the resulting color change was measured 20 s later using spectrophotometry [26]. Final values were expressed in *µ*mol · L^−1^, corresponding to the ammonia concentration in the sample.

Oxygen uptake was recorded with a portable gas analyzer (Cosmed K5, Rome, Italy) during every 30 s rest interval as well as for 2 min before and 3 min after the protocol [27]. The analyzer was calibrated prior to each session using reference gases (16% O_2_ and 5% CO_2_) and a 3 L syringe. Capillary blood lactate and glucose were measured with a Lactate Pro 2 (Arkay, Kyoto, Japan) and an Accu-Chek Aviva glucometer (Roche, Mannheim, Germany), at baseline, during rest intervals, immediately post-exercise, and every 2–3 min thereafter until values stabilized [25]. Heart rate was recorded continuously using a Polar Vantage NV transmitter (Polar Electro Oy, Kempele, Finland) synchronized with the Cosmed K5 data stream [17, 28]. Subjective effort was assessed after each stage using the Borg 6–20 Rating of Perceived Exertion scale [11], providing a perceptual complement to the objective load indicators. To ensure procedural efficiency, four researchers were assigned specific tasks during the 30-second rest intervals and the recovery duration was maintained as consistently as possible throughout the protocol.

### Data analysis

Serve precision was quantified using a point-based scale from 0–4, where 0 denoted an error (net or out) and 4 represented maximum precision (landing in the smallest target zone). Intermediate scores of 1, 2 and 3 reflected progressively higher precision across predefined serve zones [17]. Absolute frequencies of each score were recorded and converted into percentages by dividing each frequency by the total number of observations and multiplying by 100. Peak serve speed was measured for all 12 attempts per player, and mean values were calculated across attempts and between players. Precision scores were simultaneously stratified by player and attempt sequence to evaluate intra- and inter-player consistency. For temporal comparisons, serve speed and precision data were aggregated by match phases. All outputs were compiled into structured datasets and summary tables to support subsequent visualization and integrated analysis.

Oxygen uptake during exercise was estimated using a backward extrapolation method [29]. An exponential decay model was applied to the first 30 s of recovery, based on the principle that oxygen uptake peaks immediately post-exercise and then declines rapidly. A linear regression model (y = mx + b) was applied to ventilatory data, where the y-intercept represented the estimated oxygen consumption during the preceding exercise period [30]. Ventilation data were reviewed and edited to exclude irregular breathing patterns caused by coughing or signal artifacts [31]. Biochemical variables, including lactate, glucose, ammonia and additional biomarkers assessed via the Piccolo Xpress Analyzer (e.g. alanine), were extracted and organized in Microsoft Excel (version 16.0, Microsoft Corporation, Redmond, WA, USA). Data were structured by biomarker and time point to enable subsequent descriptive and comparative analysis.

### Statistical Analyses

A sample size calculation was performed a priori (GPower v. 3.1.9.7; GPower, Düsseldorf, Germany), indicating that 14 participants would provide 80% statistical power (α = 0.05) to detect a large effect size of 0.8. Data normality was assessed using the Shapiro–Wilk test. Descriptive statistics are presented as mean ± standard deviation (SD). For normally distributed data, paired-samples t-tests were used to compare pre- and post-intervention values of physiological variables. When normality assumptions were violated, the Wilcoxon signed-rank test was applied. Comparisons of lactate, glucose, oxygen uptake, heart rate, and serve speed across the 12 serve points were conducted using repeated-measures ANOVA with Bonferroniadjusted post hoc tests to control for inflation of type I error. When assumptions for parametric testing were not met, the Friedman test was used as the non-parametric alternative for repeated-measures data.

All statistical analyses were performed using SPSS software (version 29.0, IBM Corp., Armonk, NY, USA) with the significance level set at *p* ≤ 0.05. Effect sizes (ES) for pairwise comparisons were calculated using Cohen’s d and reported with 95% confidence intervals. Thresholds for interpreting d were: < 0.2 = trivial, 0.2–0.5 = small, 0.5–0.8 = moderate and > 0.8 = large. The reliability of serve speed and precision measures has been previously established with intraclass correlation coefficients ≥ 0.85–0.90 [32]. In addition, recent studies have confirmed excellent reliability of physiological and performance variables assessed under controlled exercise protocols (up to 0.95) [33].

## RESULTS

Ammonia, albumin, alanine aminotransferase, aspartate aminotransferase and creatine kinase concentrations increased (all p < 0.01), while blood urea nitrogen, sodium and calcium also showed significant elevations (p ≤ 0.05, Table 1). In contrast, total bilirubin decreased (p ≤ 0.05), whereas potassium, amylase and creatinine remained unchanged. Effect size analysis revealed large effects for ammonia, albumin, alanine aminotransferase, aspartate aminotransferase and creatine kinase, moderate effects for sodium and calcium, and a small effect for blood urea nitrogen (Table 1).

**TABLE 1:** Mean ± standard deviation values of physiological variables measured before and after the prolonged serve game, with effect sizes reported as Cohen’s *d*.

	Pre	Post	*p*	Cohen’s d
Ammonia (*μ*mol · L^−1^)	33.8 ± 7.6	111.5 ± 32.3	< 0.01**	−2.7
Blood urea nitrogen (mmol · L^−1^)	5.7 ± 0.8	6.0 ± 1.1	0.044*	−0.4
Creatinine (*μ*mol · L^−1^)	84.0 ± 53.0	97.2 ± 17.7	0.217	0.2
Total bilirubin (*μ*mol · L^−1^)	13.7 ± 10.3	13.3 ± 3.4	0.034*	−0.5
Albumin (g · L^−1^)	39.0 ± 1.4	42.0 ± 1.0	< 0.01**	−2.1
Alanine aminotransferase (U · L^−1^)	19.4 ± 4.3	23.6 ± 2.0	< 0.01**	−1.1
Aspartate aminotransferase (U · L^−1^)	33.4 ± 4.3	39.1 ± 5.9	< 0.01**	−1.7
Creatine kinase (U · L^−1^)	253.7 ± 78.2	307.2 ± 93.5	< 0.01**	−1.5
Amylase (U · L^−1^)	60.9 ± 14.7	64.6 ± 17.1	0.067	−0.4
Sodium (mmol · L^−1^)	143.1 ± 1.7	145.6 ± 3.3	0.05*	−0.7
Potassium (mmol · L^−1^)	5.4 ± 0.5	5.5 ± 0.5	0.276	−0.1
Calcium (mmol · L^−1^)	2.6 ± 0.1	2.7 ± 0.1	0.002*	−0.9

*p* ≤ 0.05 (*) and *p* < 0.01 (**) indicate differences between pre and post protocol values.

**FIG. 2:**
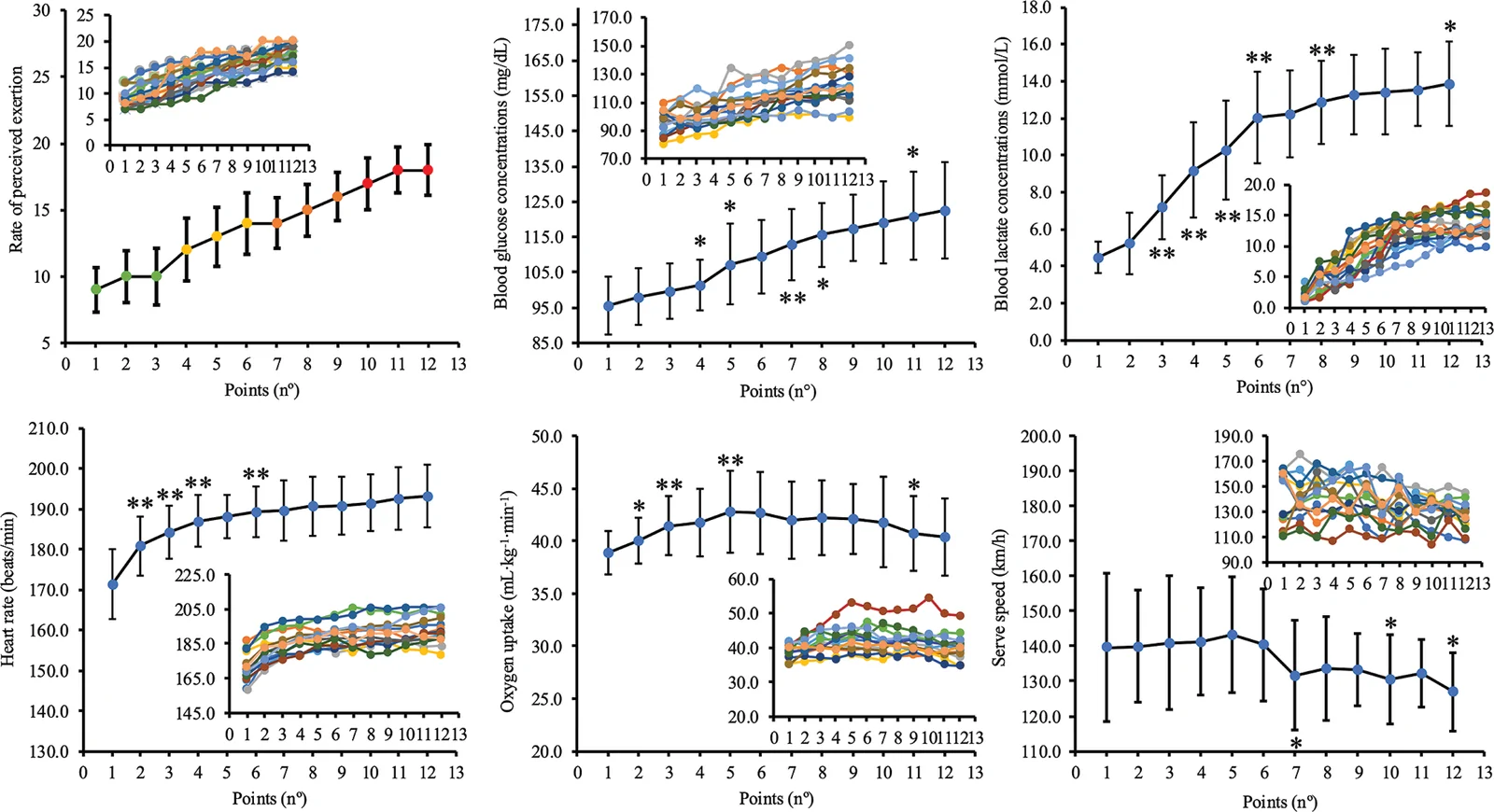
Group mean (± standard deviation) and individual values of physiological and performance variables during the prolonged service game. *p* < 0.05 (*) and *p* < 0.001 (**) indicate differences between points

Figure 2 illustrates the temporal changes in physiological and performance variables during the prolonged service game. As shown in Figure 2, ratings of perceived exertion remained stable across all points and blood glucose increased at points 4 (*p* < 0.001), 5 (*p* = 0.01), 7 (*p* = 0.006), 8 (*p* = 0.04) and 11 (*p* = 0.03). In addition, blood lactate concentrations rose from points 3–6 (*p* = 0.001 to *p* < 0.001) and again at points 8 (*p* = 0.002) and 12 (*p* = 0.003). Heart rate increased significantly from points 2–6 (*p* < 0.001 to *p* = 0.01), reaching a steady state thereafter. Oxygen uptake increased between points 2–5 (*p* = 0.040 to *p* = 0.001), plateaued until point 10, then decreased at point 11 (*p* = 0.035). Serve speed decreased at point 7 and again at points 10 and 12 (*p* = 0.02, *p* = 0.04, *p* = 0.02, Figure 2).

**FIG. 3:**
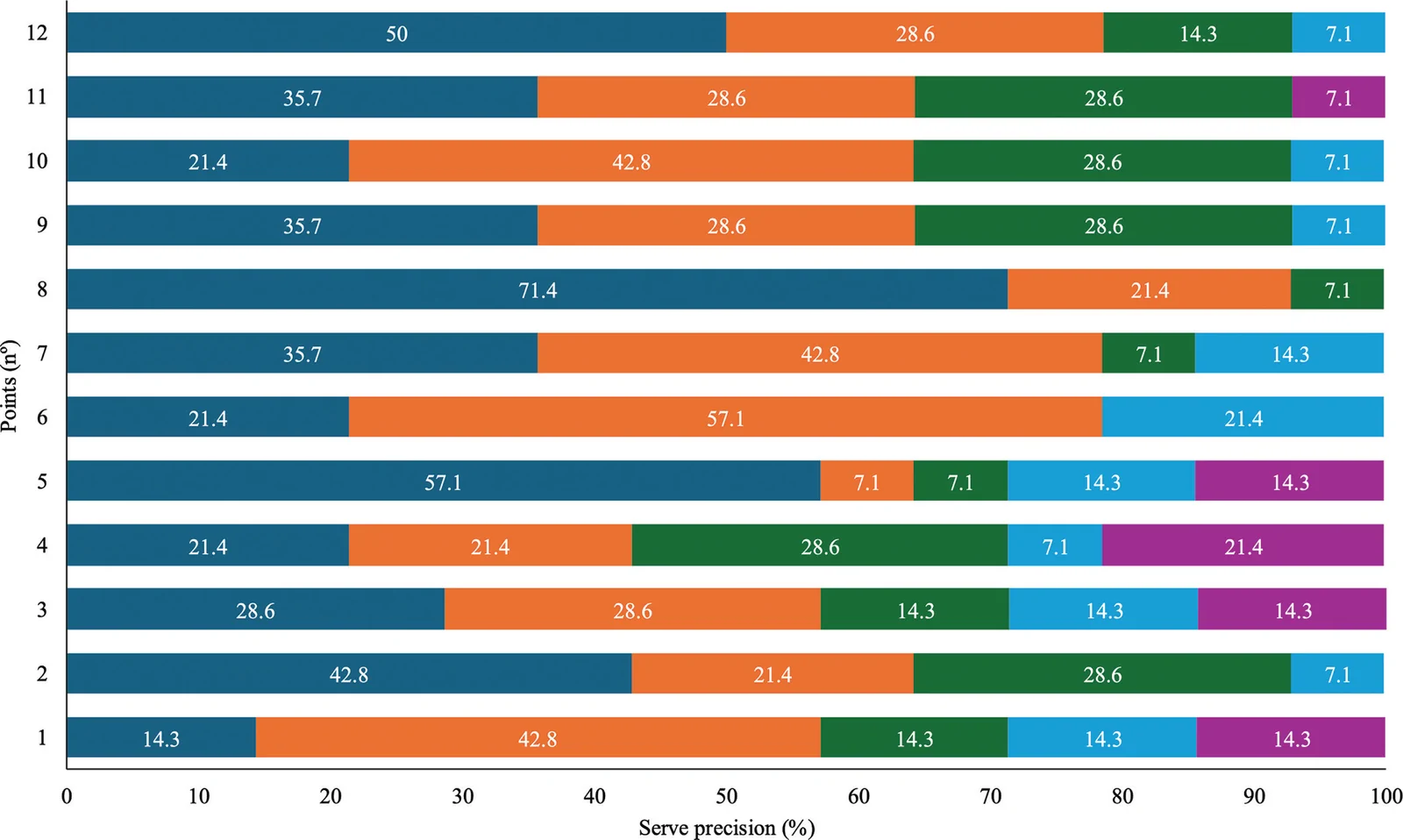
Serve precision during the prolonged service game. Dark blue, orange, green, light blue and purple represent precision scores of 0, 1, 2, 3 and 4 (respectively).

Figure 3 shows the distribution of serve precision scores throughout the prolonged service game. A marked decrease in precision occurred at point 8, where 71.4% of serves were classified as errors (score 0, Figure 3). In the earlier stages, particularly at point 5, most landed in the valid serve area (score 1), peaking at 57.1%. From point 6 onward, there was a moderate increase in precision (scores 3 and 4), although score 4 remained consistently low across the game (7.1% to 14.3%, Figure 3). Blood lactate was directly associated with perceived exertion (r = 0.609, *p* < 0.001) and heart rate (r = 0.437, *p* < 0.001). The Borg scale was positively related to glucose (r = 0.441, *p* < 0.001) and heart rate (r = 0.606, *p* < 0.001), supporting its utility as a subjective marker of fatigue. Serve precision was not directly related to physiological variables but showed a weak inverse relationship with perceived exertion (r = –0.129, *p* = 0.097).

## DISCUSSION

This study examined the physiological and performance responses of competitive tennis players during a simulated prolonged service game. The results demonstrated acute metabolic stress, evidenced by elevated concentrations of ammonia, creatine kinase, lactate and glucose, alongside electrolyte disturbances. Large effect sizes were observed for creatine kinase and ammonia, indicating substantial metabolic and muscular stress. These changes were accompanied by progressive declines in serve speed and precision, suggesting neuromuscular fatigue [4, 12, 34]. Lactate was directly related to perceived exertion, heart rate and glucose, underscoring the relationship between metabolic load and subjective fatigue. Although serve precision was not directly correlated with physiological markers, it showed an inverse association with perceived exertion, reinforcing the negative impact of fatigue on technical execution.

The experimental protocol was designed to simulate highly demanding phases of a match and to elicit meaningful physiological stress in tennis players [17, 18]. By integrating objective measures of serve speed and precision with continuous cardiovascular and metabolic monitoring, the present approach enabled a comprehensive assessment of the interaction between internal load and performance [20, 24]. The combined evaluation of heart rate, oxygen uptake and biochemical responses provided insight into the cardiovascular and systemic metabolic strain imposed by prolonged points, thereby enhancing understanding of the physiological constraints that may compromise serve performance under fatigue conditions [35].

The predominance of anaerobic metabolism, as indicated by the accumulation of metabolic by-products, may impair motor coordination and overall performance through glycolytic activation and muscular acidosis [21, 34, 35]. This metabolic shift typically occurs when phosphagen stores become depleted during repeated explosive efforts, increasing reliance on anaerobic glycolysis. Intensified glycolytic activity leads to the accumulation of lactate and hydrogen ions, lowering pH and accelerating fatigue. Concurrently, ammonia production via amino acid deamination further disrupts acid–base balance and neuromuscular control, disrupting the citric acid cycle and reducing neuromuscular efficiency [34, 35]. The observed rise in oxygen uptake may represent a limited compensatory response of the aerobic system to meet elevated energy demands [36], reinforcing the importance of improving anaerobic tolerance and buffering capacity to sustain performance during prolonged high-intensity points [8, 21].

Elevated glucose and perceived exertion reflect the substantial metabolic demands of prolonged high-intensity efforts, confirming the shift toward anaerobic energy pathways and aligning with previous tennis physiology research [14, 37]. The detection of muscular stress markers may be associated with repeated explosive actions such as the serve, with comparable fatigue alterations observed following prolonged matches [17, 38]. Moreover, electrolyte imbalances (particularly in sodium, potassium and calcium) can impair nerve conduction and muscle fiber activation [12, 35]. The protocol-induced elevations in sodium concentrations, although remaining within physiological ranges are consistent with transient hemoconcentration during high-intensity efforts in other studies. Such electrolyte shifts may contribute to acute alterations in neuromuscular function and movement efficiency during prolonged high-intensity efforts [25, 39].

Technical proficiency and neuromuscular coordination are essential for effective serve performance, which directly influences match outcomes. In the current study, speed and precision declined from the sixth point onward, indicating the accumulation of fatigue [10]. These findings suggest that physiological strain compromised serve performance, aligning with previous research showing that combined metabolic demands impair neuromuscular coordination and motor control [10, 38]. Sustained high-intensity efforts may also induce neuromuscular dysfunction, which negatively impacts tactical execution, reinforcing the importance of targeted conditioning strategies to enhance energy system efficiency and decision-making capacity.

The relationship between physiological markers and serve performance highlights the critical role of neuromuscular function and motor control in sustaining technical execution under fatigue. Serve performance is particularly susceptible due to its biomechanical complexity and reliance on precise neuromuscular timing [38]. As fatigue accumulates, elevated lactate and ammonia impair both peripheral and central mechanisms [7]. Specifically, lactate decreases intramuscular pH and disrupts excitation–contraction coupling, whereas ammonia affects brain function and motor drive, further compromising coordination. Together, these effects slow reaction time and impair motor control, both of which are essential for maintaining serve speed and precision [40]. The progressive reductions in speed and precision observed alongside physiological deterioration and decreased power support previous reports on the detrimental impact of fatigue on performance [38].

This study highlights the complex interaction between metabolic stress, neuromuscular fatigue and serve performance. Few investigations have examined tennis serve performance under physiological fatigue using such an integrated multi-marker approach. The biochemical data underscore the substantial metabolic load and muscle strain induced by repeated high-intensity efforts. These findings demonstrate how accumulated fatigue can impair serve mechanics and performance, even in experienced players. By integrating realtime performance indicators with biomarker concentrations, this study provides a robust framework for interpreting the physiological demands of a prolonged service game, offering a more comprehensive understanding of performance limitations in competitive tennis.

Some limitations should be acknowledged. First, only fourteen players were included, which restricts the generalizability of the findings to the broader tennis population. Second, age-related differences among participants may have influenced physiological responses, contributing to data variability. Third, the exclusive focus on male players limits applicability to female athletes. Fourth, testing was performed on a single court surface (grass) and under specific summer conditions, without accounting for other surfaces or environmental contexts. Fifth, the absence of hormonal markers such as cortisol and testosterone limited the assessment of stress and recovery responses. Finally, changes in biochemical and electrolyte variables were not corrected for plasma volume shifts, which may have partially influenced the magnitude of the observed post-exercise responses.

The findings of this study provide practical guidance for coaches and sports scientists aiming to optimize serve performance during prolonged service games. The observed increases in lactate, ammonia and creatine kinase reflect substantial metabolic and muscular stress, underscoring the need for structured fatigue management to preserve serve speed and precision. Training sessions may incorporate repeated high-intensity sequences with short recovery intervals, as well as serve plus shots drills performed under controlled fatigue to enhance technical consistency under stress. Rather than attempting to accelerate creatine kinase clearance, conditioning programs should progressively expose players to repeated high-intensity efforts to attenuate exercise-induced muscular stress through repeated-bout adaptations. Recovery strategies should prioritize hydration, carbohydrate availability and adequate protein intake. The integration of monitoring tools such as heart rate, ratings of perceived exertion, blood lactate, glucose and serve precision tracking may support individualized load adjustments, particularly during congested competitive periods.

Future research should examine physiological and performance responses during a real match involving multiple serve games and variable point durations to better reflect match demands. Expanding the protocol to include additional tennis actions, (e.g. net approaches) would enhance ecological validity. Longitudinal investigations assessing chronic adaptations to serve fatigue training may further clarify the long-term metabolic and neuromuscular implications of repeated high-intensity match scenarios. Moreover, studies including larger sample sizes, female players, and different competitive contexts such as doubles match play would contribute to a broader and more comprehensive understanding of fatigue responses in tennis [4].

## CONCLUSIONS

This study demonstrated that a simulated prolonged service game induces physiological stress and impairs performance in competitive tennis players. Repeated serves and subsequent shots led to marked increases in metabolic and muscular stress markers, accompanied by elevated cardiovascular strain. These internal responses were associated with reductions in serve speed and fluctuations in precision, confirming the negative influence of accumulated fatigue on technical execution. The intra-subject design revealed interindividual variability, highlighting that players exhibit distinct physiological and performance responses to this protocol. Collectively, these findings emphasize the serve as a critical phase in tennis performance and support the implementation of targeted conditioning, fatigue management and monitoring strategies to enhance physiological resilience and maintain technical stability under demanding competitive conditions.

## References

[1] Fernandez-Fernandez J, Ulbricht A, Ferrauti A. Fitness testing of tennis players: How valuable is it? Br J Sports Med. 2014; 48(Suppl 1):i22–i31. 24668375 10.1136/bjsports-2013-093152 PMC3995228

[2] Yang W, Yin L, Poon ET, Kit Ho IM, Liu H, Qi B, et al. Effects of low-volume court-based sprint interval training on aerobic capacity and sport-specific endurance performance in competitive tennis players. Biol Sport. 2025; 42(1):223–32. 10.5114/biolsport.2025.139088 PMC11694208 39758185

[3] Fernandez J, Mendez-Villanueva A, Pluim BM. Intensity of tennis match play. Br J Sports Med. 2006; 40(5):387–91; discussion 91. 16632566 10.1136/bjsm.2005.023168 PMC2653872

[4] Pluim B, Jansen M, Williamson S, Berry C, Camporesi S, Fagher K, et al. Physical Demands of Tennis Across the Different Court Surfaces, Performance Levels and Sexes: A Systematic Review with Meta-analysis. Sports Med. 2023; 53. 10.1007/s40279-022-01807-8 36752978

[5] Gomes RV, Moreira A, Lodo L, Nosaka K, Coutts AJ, Aoki MS. Monitoring training loads, stress, immune-endocrine responses and performance in tennis players. Biol Sport. 2013; 30(3):173–80. 24744485 10.5604/20831862.1059169 PMC3944572

[6] Szpala A, Rutkowska-Kucharska A, Syrewicz P. The assessment of specific physical fitness of children aged 8 and 9 years participating in tennis classes using the Jindrich Hoehm test. Biomed Hum Kinet. 2014; 6.

[7] Kovacs MS. Applied physiology of tennis performance. Br J Sports Med. 2006; 40(5):381–5; discussion 6. 16632565 10.1136/bjsm.2005.023309 PMC2653871

[8] Morais JE, Bragada JA. Relationship between Oxygen Uptake Reserve and Heart Rate Reserve in Young Male Tennis Players: Implications for Physical Fitness Monitoring. Int J Environ Res Public Health. 2022; 19(23). 10.3390/ijerph192315780 PMC9735773 36497853

[9] Tóth PJ, Csáki I, Négyesi J, Dobos K, Havanecz K, Sáfár S, et al. External training load and rating of perceived exertion comparison between different playing styles and winning vs. losing matches in elite tennis. Front Sports Act Living. 2025; 7:1613661. 40666312 10.3389/fspor.2025.1613661 PMC12259623

[10] Bilić Z, Sinković F, Barbaros P, Novak D, Zemková E. Exercise-Induced Fatigue Impairs Change of Direction Performance and Serve Precision among Young Male Tennis Players. Sports. 2023; 11. 10.3390/sports11060111 PMC10302430 37368561

[11] Mendez-Villanueva A, Fernandez-Fernández J, Bishop D, Fernandez-Garcia B. Ratings of perceived exertion-lactate association during actual singles tennis match play. J Strength Cond Res. 2010; 24(1):165–70. 19625975 10.1519/JSC.0b013e3181a5bc6d

[12] Reid M, Duffield R. The development of fatigue during match-play tennis. Br J Sports Med. 2014; 48 Suppl 1(Suppl 1):i7–11. 24668384 10.1136/bjsports-2013-093196 PMC3995216

[13] Knittel M, Guszkowska M. Strategies of coping with stress and the sport results of alpine skiers and tennis players. Biomed Hum Kinet. 2016; 8.

[14] Kovacs M. Tennis physiology: training the competitive athlete. Sports Med. 2007; 37:189–98. 17326695 10.2165/00007256-200737030-00001

[15] Cádiz Gallardo MP, Pradas de la Fuente F, Moreno-Azze A, Carrasco Páez L. Physiological demands of racket sports: a systematic review. Front Psychol. 2023; 14. 10.3389/fpsyg.2023.1149295 PMC10101231 37063547

[16] Brito AV, Fonseca P, Costa MJ, Cardoso R, Santos CC, Fernandez-Fernandez J, et al. The Influence of Kinematics on Tennis Serve Speed: An In-Depth Analysis Using Xsens MVN Biomech Link Technology. Bioeng. 2024; 11(10). 10.3390/bioengineering11100971 PMC11504545 39451347

[17] Rota S, Morel B, Saboul D, Rogowski I, Hautier C. Influence of fatigue on upper limb muscle activity and performance in tennis. J Electromyogr Kinesiol. 2014; 24(1):90–7. 24239164 10.1016/j.jelekin.2013.10.007

[18] Brito AV, Afonso J, Silva G, Fernandez-Fernandez J, Fernandes RJ. Biophysical characterization of the tennis serve: A systematic scoping review with evidence gap map. J Sci Med Sport. 2024; 27(2):125–40. 37980182 10.1016/j.jsams.2023.10.018

[19] Fernandez-Fernandez J, Kinner V, Ferrauti A. The physiological demands of hitting and running in tennis on different surfaces. J Strength Cond Res. 2010; 24(12):3255–64. 21088546 10.1519/JSC.0b013e3181e8745f

[20] Brito AV, Carvalho DD, Fonseca P, Monteiro AS, Fernandes A, Fernández-Fernández J, et al. Shoulder Torque Production and Muscular Balance after Long and Short Tennis Points. Int J Environ Res Public Health. 2022; 19(23). 10.3390/ijerph192315857 PMC9737849 36497932

[21] Reid MM, Duffield R, Minett GM, Sibte N, Murphy AP, Baker J. Physiological, perceptual, and technical responses to on-court tennis training on hard and clay courts. J Strength Cond Res. 2013; 27(6):1487–95. 22890497 10.1519/JSC.0b013e31826caedf

[22] Qureshi H, Omer A, Khan A, Rauf U, Ahmad U, Rehman M. The Effects Of Functional Kinesio Taping On Acromio Humeral Distance, Pain, Quality Of Life And Disability In Overhead Athletes With Rotator Cuff Tendinopathy: A Randomized Control Trial. Advances in Rehabilitation. 2024; 38.

[23] Baiget E, Corbi F, López JL. Influence of anthropometric, ball impact and landing location parameters on serve velocity in elite tennis competition. Biol Sport. 2023; 40(1):273–81. 36636186 10.5114/biolsport.2023.112095 PMC9806764

[24] Carboch J, Süss V, Kocib T. Ball machine usage in tennis: movement initiation and swing timing while returning balls from a ball machine and from a real server. J Sports Sci Med. 2014; 13(2):304–8. 24790483 PMC3990883

[25] Murata K, Glaser L, Nardiello M, Richardson S, Ramanathan LV, Carlow DC. Analytical performance of the Abaxis Piccolo Xpress^®^ point of care analyzer in whole blood, serum, and plasma. Clin Biochem. 2015; 48(18):1344–6. 26255121 10.1016/j.clinbiochem.2015.08.002

[26] Arkray I. PocketChem BA PA-4140 Instruction Manual. Kyoto, Japan; 2022. Report No.: B120820–01D.

[27] Cardoso F, Costa MJ, Colaço P, Vilas-Boas JP, Pinho JC, Pyne DB, et al. Ventilatory and Perceived Ergogenic Effects of Mandibular Forward Repositioning During Running at Maximal Oxygen Uptake Intensity. J Strength Cond Res. 2025; 39(1):e13–e9. 39303201 10.1519/JSC.0000000000004953

[28] Cardoso F, Carvalho DD, Cardoso R, Maligno F, Vilas-Boas JP, Pinho JC, et al. Mandibular repositioning effects on running until exhaustion at moderate intensity. J Sports Med Phys Fitness. 2025. 10.23736/S0022-4707.25.16798-4 40742788

[29] Monteiro AS, Azevedo RMS, Zacca R, Ogonowska-Slodownik A, Buzzachera CF, Vilas-Boas JP, et al. VO(2)FITTING Software: New Insights and Practical Applications (VO(2)FITTING Software Update). J Hum Kinet. 2025; 97:89–100. 40463317 10.5114/jhk/194124 PMC12127917

[30] Monteiro AS, Galano JP, Cardoso F, Buzzachera CF, Vilas-Boas JP, Fernandes RJ. Kinematical and Physiological Responses of Overground Running Gait Pattern at Different Intensities. Sensors (Basel). 2024; 24(23). 10.3390/s24237526 PMC11644620 39686063

[31] Cardoso F, Monteiro AS, Vilas-Boas JP, Pinho JC, Pyne DB, Fernandes RJ. Effects of Wearing a 50% Lower Jaw Advancement Splint on Biophysical and Perceptual Responses at Low to Severe Running Intensities. Life. 2022; 12(2):253. 35207540 10.3390/life12020253 PMC8875792

[32] González-González I, Rodríguez-Rosell D, Clavero-Martín D, Mora-Custodio R, Pareja-Blanco F, García JMY, et al. Reliability and Accuracy of Ball Speed During Different Strokes in Young Tennis Players. Sports Med Int Open. 2018; 2(5):E133–e41. 30539130 10.1055/a-0662-5375 PMC6259460

[33] Cardoso R, Rios M, Fonseca P, Leão J, Cardoso F, Abraldes JAA, et al. Assessment of Angular and Straight Linear Rowing Ergometers at Different Intensities of Exercise. Sensors. 2024; 24(17):5686. 39275598 10.3390/s24175686 PMC11397995

[34] Fernandez-Fernandez J, Sanz D, Mendez-Villanueva A. A Review of the Activity Profile and Physiological Demands of Tennis Match Play. Strength Cond J. 2009; 31:15–26. 10.1519/JSC.0b013e318194208a 19197208

[35] Bergeron M, Maresh C, Armstrong L, Signorile J, Castellani J, Kenefick R, et al. Fluid-Electrolyte Balance Associated with Tennis Match Play in a Hot Environment. Int J Sport Nutr Exerc Metab. 1995; 5:180–93. 10.1123/ijsn.5.3.180 8547936

[36] Murias J, Lanatta D, Arcuri C, Laíño F. Metabolic and functional responses playing tennis on different surfaces. J Strength Cond Res. 2007; 21:112–7. 17313272 10.1519/00124278-200702000-00021

[37] Martin C, Thevenet D, Zouhal H, Mornet Y, Delès R, Crestel T, et al. Effects of playing surface (hard and clay courts) on heart rate and blood lactate during tennis matches played by high-level players. J Strength Cond Res. 2011; 25(1):163–70. 21157392 10.1519/JSC.0b013e3181fb459b

[38] Martin C, Bideau B, Delamarche P, Kulpa R. Influence of a Prolonged Tennis Match Play on Serve Biomechanics. PLoS One. 2016; 11(8):e0159979. 27532421 10.1371/journal.pone.0159979 PMC4993075

[39] Komka Z, Szilágyi B, Molnár D, Sipos B, Tóth M, Sonkodi B, et al. Exercise-related hemoconcentration and hemodilution in hydrated and dehydrated athletes: An observational study of the Hungarian canoeists. PLoS One. 2022; 17(12):e0277978. 36584041 10.1371/journal.pone.0277978 PMC9803156

[40] Lambrich J, Muehlbauer T. Effects of fatigue on physiological, physical fitness, and stroke performance related parameters in healthy tennis players: a systematic review and meta-analysis. Front Sports Act Living. 2025; 7:1578914. 40365546 10.3389/fspor.2025.1578914 PMC12069318

